# Supplementary material for: The Long-Term Persistence of Borrelia burgdorferi Antigens and DNA in the Tissues of a Patient with Lyme Disease
Source: Antibiotics (Basel). 2019 Oct 11;8(4):183. doi: 10.3390/antibiotics8040183 (PMC6963883; doi:10.3390/antibiotics8040183)
Supplement: Supplementary file 1 [file antibiotics-08-00183-s001.pdf]

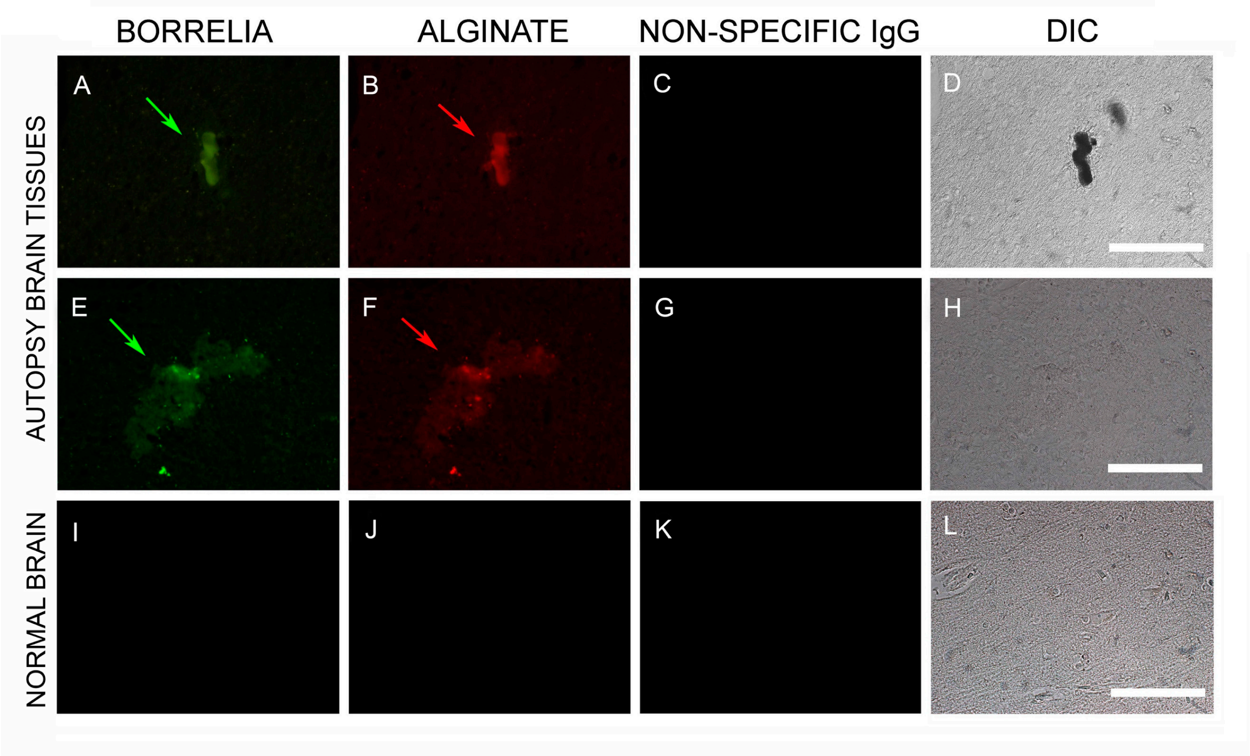

Figure S1. Representative images of IHC staining of brain autopsy tissues with *Borrelia* and alginate specific antibodies. Sections containing *Borrelia* positive aggregates (green staining: Panels **A** and **E**) were further analyzed for alginate presence using a sequential section stained with alginate-specific antibody (red staining: Panels **B** and **F**) respectively. As a negative control, normal human brain tissue was stained with the same *Borrelia* and alginate antibodies (Panels **I** and **J**). Non-specific IgG antibody was used as an additional negative control in the infected and normal tissues (Panels **C, G,** and **K O**). Differential interference microscopy (DIC) showing the size and tissue morphology of the tissues (Panels **D, H,** and **L**). Scale bars: 200 µm.

Antibiotics **2019**, *8*, 183; doi:10.3390/antibiotics8040183 www.mdpi.com/journal/antibiotics

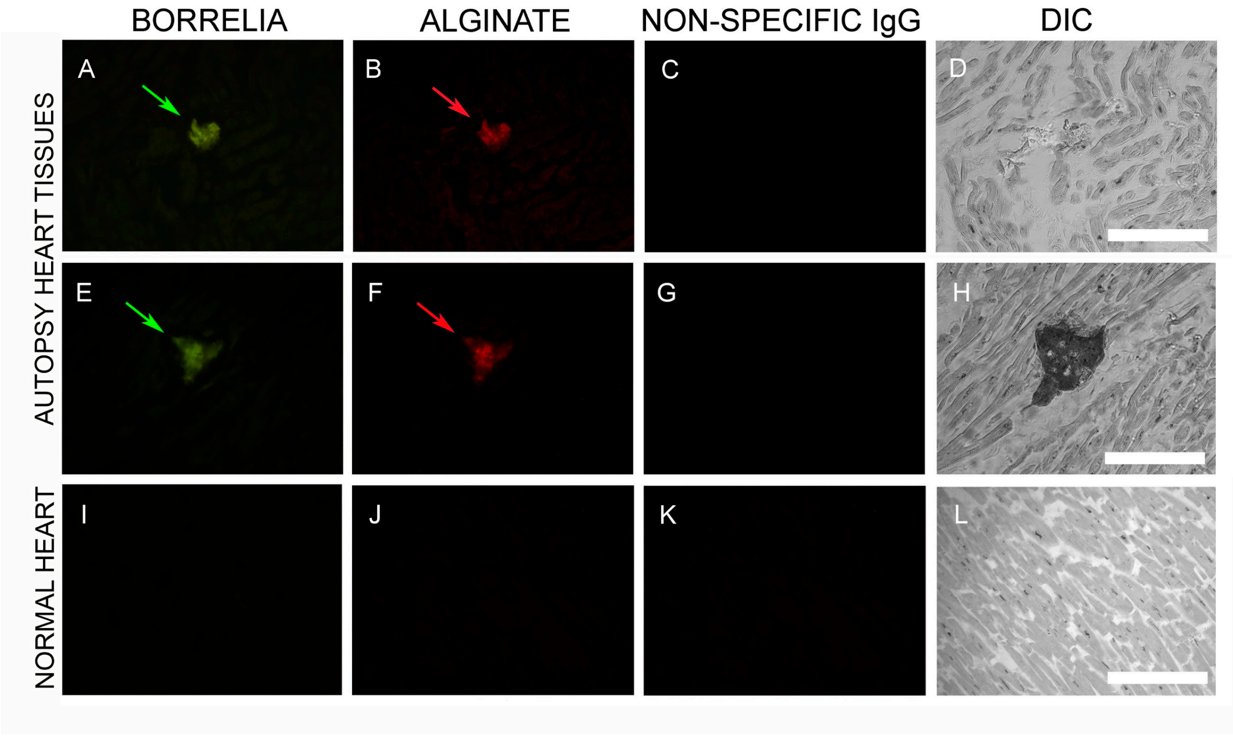

Figure S2. Representative images of IHC staining of heart tissues with *Borrelia* and alginate specific antibodies**.** Sections containing *Borrelia* positive aggregates (green staining: Panels **A** and **E**) were further analyzed for alginate presence using a sequential section stained with alginate-specific antibody (red staining: Panels **B** and **F**) respectively. As a negative control, normal human brain tissue was stained with the same *Borrelia* and alginate antibodies (Panels **I** and **J**). Non-specific IgG antibody was used as an additional negative control in the infected and normal tissues (Panels **C, G,** and **K O**). Differential interference microscopy (DIC) showing the size and tissue morphology of the tissues (Panels **D, H,** and **L**). Scale bars: 200 µm.

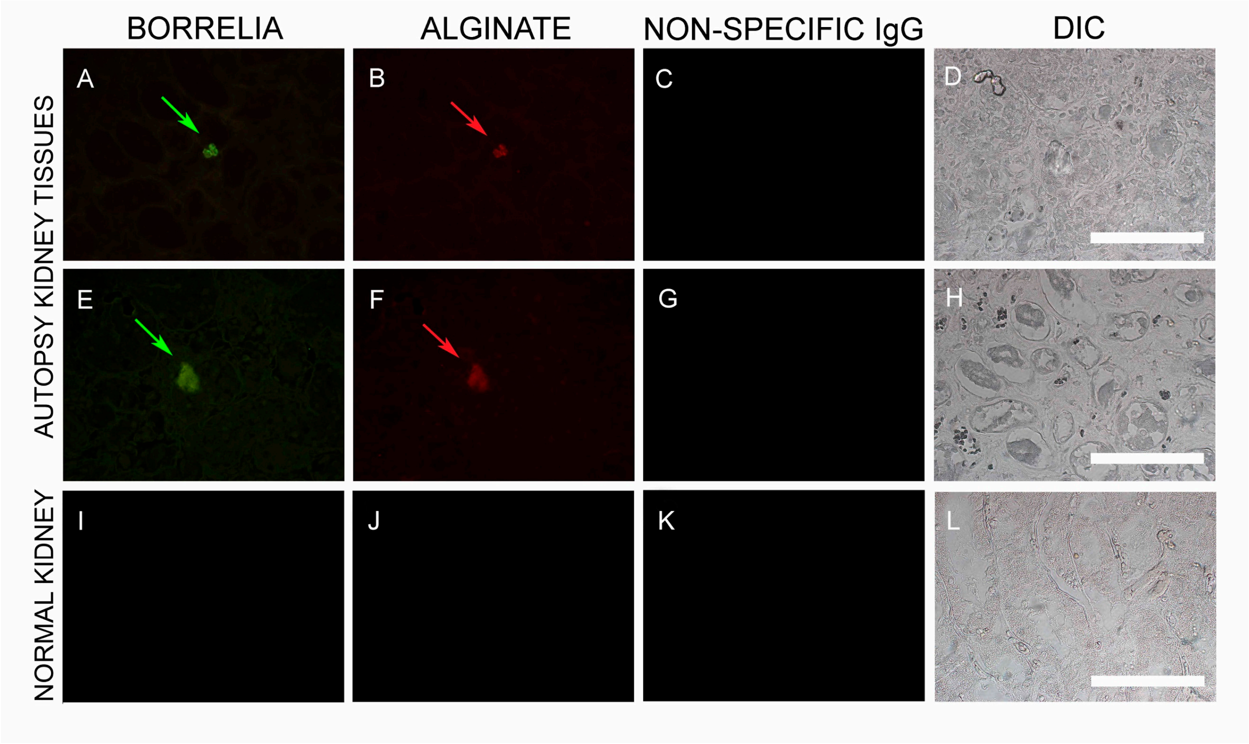

Figure S3. Representative images of IHC staining of kidney tissues with *Borrelia* and alginate specific antibodies. Sections containing *Borrelia* positive aggregates (green staining: Panels **A** and **E**) were further analyzed for alginate presence using a sequential section stained with alginate-specific antibody (red staining: Panels **B** and **F**) respectively. As a negative control, normal human brain tissue was stained with the same *Borrelia* and alginate antibodies (Panels **I** and **J**). Non-specific IgG antibody was used as an additional negative control in the infected and normal tissues (Panels **C, G,** and **K O**). Differential interference microscopy (DIC) showing the size and tissue morphology of the tissues (Panels **D, H,** and **L**). Scale bars: 200 µm.

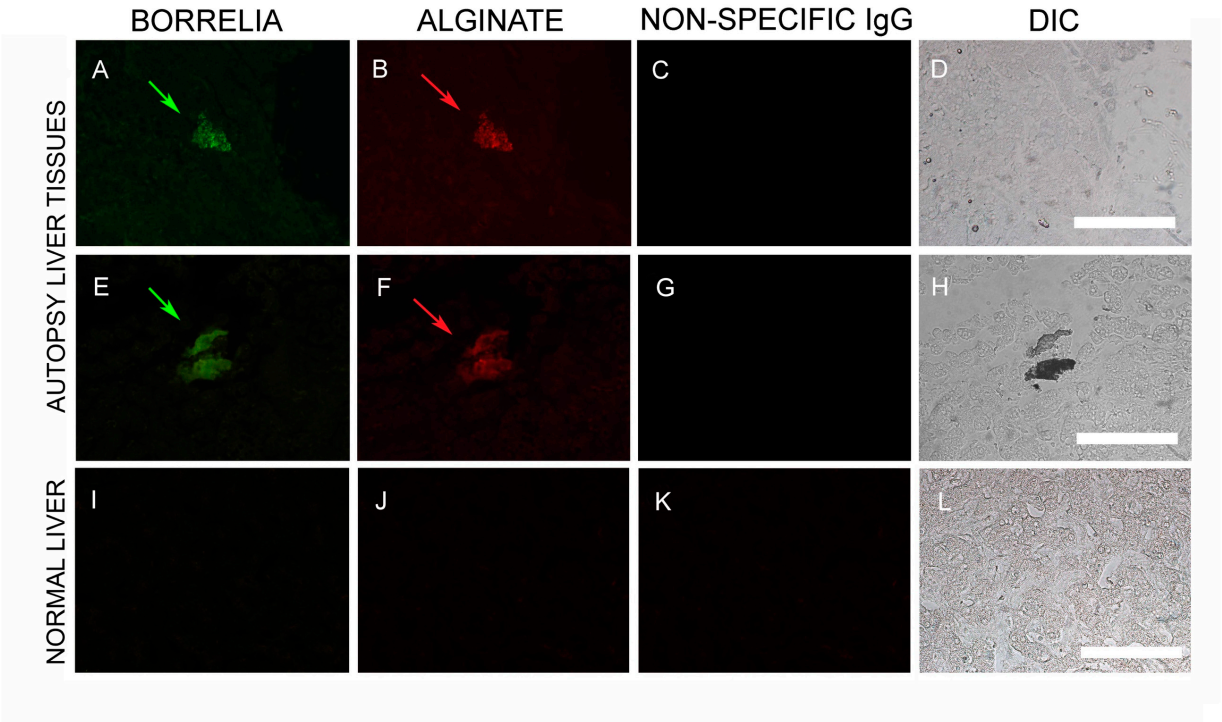

Figure S4. Representative images of IHC staining of liver tissues with *Borrelia* and alginate specific antibodies. Sections containing *Borrelia* positive aggregates (green staining: Panels **A** and **E**) were further analyzed for alginate presence using a sequential section stained with alginate-specific antibody (red staining: Panels **B** and **F**) respectively. As a negative control, normal human brain tissue was stained with the same *Borrelia* and alginate antibodies (Panels **I** and **J**). Non-specific IgG antibody was used as an additional negative control in the infected and normal tissues (Panels **C, G,** and **K O**). Differential interference microscopy (DIC) showing the size and tissue morphology of the tissues (Panels **D, H,** and **L**). Scale bars: 200 µm.

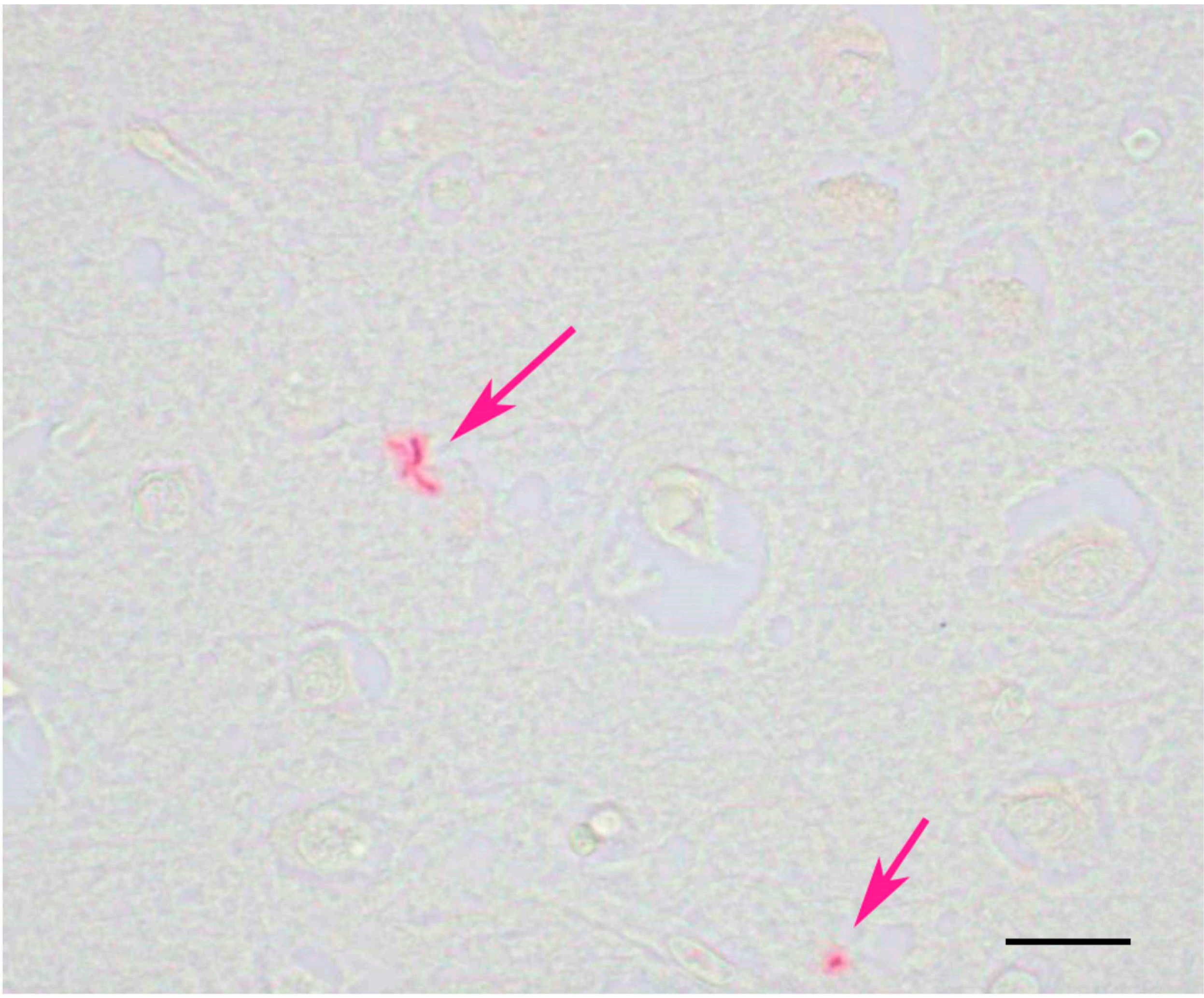

Figure S5. Representative image of IHC staining of a brain tissue for *B. burgdorferi* (red staining with red arrows) at the site of cerebral vasculitis. The IHC was performed at the Innsbruck Medical Center as described previously (x). Scale bar: 10 µm.
